# Maternal left ventricular function and adverse neonatal outcomes in women with cardiac disease

**DOI:** 10.1007/s00404-022-06635-9

**Published:** 2022-06-03

**Authors:** Elizabeth J. Eggleton, Catriona J. Bhagra, Charlotte J. Patient, Mark Belham, Janet Pickett, Catherine E. Aiken

**Affiliations:** 1 The Medical School, Newcastle University, Framlington Place, Newcastle Upon Tyne, NE2 4HH UK; 2 Cambridge University Hospitals NHS Foundation Trust, Hills Road, Cambridge, CB2 0SW UK; 3 Department of Obstetrics and Gynaecology, The Rosie Hospital and NIHR Cambridge Biomedical Research Centre, University of Cambridge, Box 223, Cambridge, CB2 0SW UK

**Keywords:** Pregnancy, Neonatal outcomes, Cardiac disease, Cardiomyopathy, Left ventricular function, Global longitudinal strain

## Abstract

**Purpose:**

To evaluate the relationship between maternal left ventricular systolic function, utero-placental circulation, and risk of adverse neonatal outcomes in women with cardiac disease.

**Methods:**

119 women managed in the pregnancy heart clinic (2019–2021) were identified. Women were classified by their primary cardiac condition. Adverse neonatal outcomes were: low birth weight (< 2500 g), small-for-gestational-age (< 10th birth-weight centile), pre-term delivery (< 37 weeks’ gestation), and fetal demise (> 20 weeks’ gestation). Parameters of left ventricular systolic function (global longitudinal strain, radial strain, ejection fraction, average S’, and cardiac output) were calculated and pulsatility index was recorded from last growth scan.

**Results:**

Adverse neonatal outcomes occurred in 28 neonates (24%); most frequently in valvular heart disease (*n* = 8) and cardiomyopathy (*n* = 7). Small-for-gestational-age neonates were most common in women with cardiomyopathy (*p* = 0.016). Early pregnancy average S’ (*p* = 0.03), late pregnancy average S’ (*p* = 0.02), and late pregnancy cardiac output (*p* = 0.008) were significantly lower in women with adverse neonatal outcomes than in those with healthy neonates. There was a significant association between neonatal birth-weight centile and global longitudinal strain (*p* = 0.04) and cardiac output (*p* = 0.0002) in late pregnancy. Pulsatility index was highest in women with cardiomyopathy (*p* = 0.007), and correlated with average S’ (*p* < 0.0001) and global longitudinal strain (*p* = 0.03) in late pregnancy.

**Conclusion:**

Women with cardiac disease may not tolerate cardiovascular adaptations required during pregnancy to support fetal growth. Adverse neonatal outcomes were associated with reduced left ventricular systolic function and higher pulsatility index. The association between impaired systolic function and reduced fetal growth is supported by insufficient utero-placental circulation.

**Supplementary Information:**

The online version contains supplementary material available at 10.1007/s00404-022-06635-9.

## Introduction

Meeting the physiological demands of pregnancy requires considerable adaptation of the maternal cardiovascular system [1]. The maternal vasculature must accommodate a significant increase in blood volume, increasing both preload and stroke volume [1, 2], and a concomitant drop in total peripheral resistance. Maternal cardiac output increases by up to 50% during pregnancy [3–5], with the consequent altered loading of the left ventricle resulting in reversible cardiac remodelling [6]. It has been estimated that left ventricular end-diastolic diameter increases by 7–12% and left ventricular mass increases by up to 50% in healthy pregnancy [1, 3, 4, 6–8]. These cardiovascular adaptations are primarily hormonally mediated and are necessary to ensure sufficient utero-placental circulation for fetal growth and development [1, 5, 9].

1–4% of pregnancies globally are complicated by maternal cardiac disease, which accounts for a high percentage of maternal deaths [10, 11]. Maternal cardiac disease is the leading cause of indirect maternal deaths in the United Kingdom [12] and of maternal death in the United States [13]. The association between cardiac disease in pregnancy and adverse maternal outcomes is well established [10, 14–19]; however, neonatal outcomes are less well studied. Pregnant women with cardiac disease may have impaired maternal cardiovascular adaptation to pregnancy and hence impaired utero-placental circulation, potentially leading to an increased risk of adverse neonatal outcomes [20–22]. Evidence suggests reduced maternal left ventricular systolic function during pregnancy in women with heart disease leads to placental insufficiency and hence prevents the fetus from fulfilling its growth potential [17, 20, 21, 23]. However, it is not currently known which parameters of left ventricular function may best predict sub-optimal fetal growth.

Assessment of left ventricular function is often limited to an estimate of ejection fraction from transthoracic echocardiograms [24]. Two-dimensional speckle tracking echocardiography is a newer imaging technique, which allows segmental myocardial deformation analysis of the left ventricle to be calculated as global longitudinal strain (GLS) [25, 26]. GLS is an accurate and reproducible parameter to assess myocardial systolic function outside of pregnancy that allows subclinical dysfunction to be identified [27, 28]. However, studies have found mixed results regarding changes in left ventricular parameters, such as GLS, in healthy pregnancy and expected normal ranges have not been established [4, 6, 29, 30]. There is currently a lack of evidence to evaluate whether strain analysis would be a useful additional assessment to predict pregnancy outcomes in women with heart disease [31].

The aim of this study was to evaluate which parameters of left ventricular systolic function are most closely associated with risk of adverse neonatal outcomes in the context of maternal cardiac disease. Moreover, to assess whether the addition of strain calculations could improve prediction of adverse outcomes. The relationship between maternal cardiac function and utero-placental flow in women with cardiac disease will be explored to investigate the relationship with fetal growth. Better understanding of the relationship between maternal left ventricular systolic function, utero-placental flow, and adverse neonatal outcomes will allow for improvements in clinical management, such as closer fetal growth surveillance for pregnancies at highest risk.

## Materials and methods

All women with cardiac disease that attended the pregnancy heart clinic at a single tertiary UK obstetrics centre between January 2019 and October 2021 were screened against inclusion criteria (Online Resource 1). 181 women attended the service, of whom 119 (66%) were eligible for inclusion in the analytic cohort (Online Resource 2).

### Data extraction

Maternal demographic data were abstracted from electronic medical records; including maternal age (at time of delivery), pre-pregnancy BMI, parity status (prior to delivery), cardiac history, smoking status, cardiac medications, and comorbidities. The clinical history of the patient’s cardiac condition was recorded and used to classify women into one of six groups based on their primary cardiac condition (aortopathy, arrhythmia, cardiomyopathy, congenital heart disease, valvular heart disease, and other), and detailed subtyping was performed (Online Resource 3).

Data on neonatal outcomes were collected from maternal delivery records and the neonatal medical record; including mode of delivery, gestational age at delivery, birth weight, and sex. Birth-weight centile was calculated using Intergrowth-21 standards [32]. Adverse neonatal outcome were pre-defined as: low birth weight (< 2500 g), small-for-gestational-age (< 10th birth-weight centile), premature delivery (< 37 weeks’ gestation), intrauterine fetal death (death > 20 weeks’ gestation), and neonatal death (death within the first 30 days of delivery).

Umbilical artery Doppler pulsatility index (PI) was recorded from the last obstetric ultrasound scan prior to birth (gestational age range 30–37 weeks). The PI did not vary significantly with gestational age within this range.

### Calculation of echocardiographic parameters

Echocardiographic studies were identified for each participant and classified as: (i) pre-pregnancy (within 5 years of pregnancy), (ii) early pregnancy (0–28 weeks), and (iii) late pregnancy (> 28 weeks). All echocardiograms were performed in the left lateral decubitus position using GE Healthcare machines and a 2.5-MHz transducer. Parameters of left ventricular systolic function were calculated: GLS, radial strain at papillary muscle level (RS), ejection fraction (EF; Biplane Simpson’s method), averaged peak longitudinal myocardial velocities of the lateral and septal mitral valve annulus (Average S’), and cardiac output.

Two-dimensional speckle tracking echocardiography technique was used to calculate GLS and RS (Online Resource 4). Offline strain analysis was performed using EchoPAC (GE Healthcare; calculations performed according to the methods specified in Online Resource 5). A frame rate of 40–90 frames per second was required for analysis [33, 34]. Studies without required images, images with poor myocardial definition, or significant heart rate variability were excluded (Online Resource 6).

All strain measurements were performed by a single trained observer. To assess intra-observer variability of GLS and RS, repeat measurements were performed 9 months after initial measurements on 25 randomly chosen echocardiograms and intra-class correlation coefficients (ICC) were calculated. The repeatability of the measurements was strong for both GLS (ICC 0.93, 95% CI 0.82–0.98) and RS (ICC 0.76, 95% CI 0.43–0.91). Inter-observer variability was assessed using GLS measurements performed by further 3 independent accredited echocardiographers using blinded images from 5 echocardiograms (ICC 0.89, 95% CI 0.65–0.99).

Cardiac output was calculated as the product of stroke volume and heart rate. Stroke volume was calculated using the cross-sectional area of the left ventricular outflow tract measured in the parasternal long axis in systole and the velocity time integral of the pulsed wave Doppler waveform measured in the five-chamber or three-chamber view. Images with poor Doppler alignment were excluded. Heart rate was averaged from 4 images obtained during the echocardiogram.

### Statistical analysis

The Kolmogoroff–Smirnoff test was used to assess normality of the distribution of data. Continuous data are reported as mean and standard deviation (SD) or median with interquartile range (IQR), depending on the distribution. Categorical data are presented as absolute numbers and percentages. Univariate analyses were performed to compare demographics between women with adverse neonatal outcomes and no adverse neonatal outcomes. Intergroup comparison was performed using one-way ANOVA, Student’s *t *test, or the Mann–Whitney test for numerical data and Pearson’s chi-squared test for categorical data. Correlations between numeric variables were assessed using linear regression models. The association between adverse neonatal outcomes and each parameter of left ventricular systolic function was assessed using binomial logistic regression models, with and without adjustment for beta-blockade. All data analysis was performed using GraphPad Prism (v9.2.0) and R statistical software (v4.1.1). A two-tailed p value of 0.05 was considered statistically significant.

## Results

### Neonatal outcomes

Adverse neonatal outcomes occurred in 28/119 (24%) pregnancies (Table 1). Premature delivery (*n* = 19/119, 16%) and low birth weight (*n* = 19/119, 16%) were the most common neonatal complications (Table 1). Of the 19 premature infants, 47% (9/19) were iatrogenic early deliveries due to maternal cardiac disease (Table 1). 20% (24/119) women required hospital admission during pregnancy due to their cardiac condition; 67% (16/24) of these women had an adverse neonatal outcome (Table 1). Vaginal delivery was the most frequent mode of delivery (60%; Table 1). The median gestational age at delivery was 39 weeks (IQR 37–39 weeks) and median birth weight was 3090 g (IQR 2680–3400 g; Table 1).

**Table 1:** Details of adverse neonatal outcomes and delivery details in women with cardiac disease

Outcome	All eligible women (*n* = 119)
Adverse neonatal outcomes	28 (24%)
Premature delivery	19 (16%)
Low birth weight	19 (16%)
Small-for-gestational-age	9 (8%)
Intrauterine or neonatal death	≤ 3
Delivered < 37 weeks for cardiac reason	9 (8%)
Mode of delivery	
Vaginal	71 (60%)
Spontaneous vaginal delivery	25
Induction of labour	46
Caesarean section	48 (40%)
Elective caesarean section	32
Emergency caesarean section	16
Gestational age (weeks)	39 (37–39)
Birth weight (grams)	3090 (2680–3400)
Birth-weight centile	45.32 (27.35–72.68)

Data presented as *n* (%) and median (IQR)

Small number suppression applied to cells with three or fewer women

*IQR* Interquartile range

There were no significant differences in the maternal characteristics of women who experienced adverse neonatal outcomes compared to those with healthy neonates (Table 2).

**Table 2:** Maternal demographic data and baseline medical history and univariate analysis of potential predictors of adverse neonatal outcomes

Characteristic	All patients (*n* = 119)	Adverse neonatal outcomes (*n* = 28)	No adverse neonatal outcomes (*n* = 91)	*P* value
Maternal age (years)	31.2 ± 5.8	30.9 ± 5.7	31.3 ± 5.8	0.70
Maternal BMI (kg/m^2^)	28.06 ± 6.8	28.13 ± 6.5	28.01 ± 6.9	0.94
Comorbidities				
Diabetes in pregnancy	9	≤ 3	6	
Respiratory disease	12	≤ 3	9	
Hypertension	17	7	10	
Other maternal condition	10	5	5	
Beta-blockers	54 (45%)	18 (64%)	36 (40%)	**0.03**
Smoking during/prior to pregnancy	17	≤ 3	14	0.54
Parity				
0	67	17	50	
1	28	5	23	
≥ 2	24	6	18	
Umbilical artery PI	0.91 ± 0.18	1.05 ± 0.24	0.87 ± 0.13	** < 0.0001**

Data presented as n (%) or mean ± SD

Small number suppression applied to cells with three or fewer women

Bold values indicate the statistically significant results

*BMI* Body mass index, *PI* Pulsatility index, *SD* Standard deviation

Maternal left ventricular function by lesion type was characterised (Online Resource 7). Adverse neonatal outcomes occurred most frequently in women with cardiomyopathy and valvular heart disease (*n* = 15/119, 12.6%; Table 3) compared to other forms of heart disease. Small-for-gestational-age infants were significantly more likely in women with cardiomyopathy compared to women with other cardiac conditions (56% vs 44%, *p* = 0.02; Table 3).

**Table 3:** Breakdown of neonatal outcomes and umbilical artery pulsatility index by maternal cardiac condition

Neonatal outcomes	Aortopathy (*n* = 8)	Arrhythmia (*n* = 24)	CMP (*n* = 26)	CHD (*n* = 28)	VHD (*n* = 17)	Other (*n* = 16)
Gestational age (weeks)	38.73 ± 0.59	38.42 ± 3.39	37.93 ± 1.32	38.73 ± 1.75	38.02 ± 2.56	38.30 ± 2.72
Birth weight (grams)	3323 ± 556	3098 ± 705	2921 ± 493	3064 ± 416	2886 ± 663	3173 ± 820
Umbilical artery PI	0.82 ± 0.07	0.85 ± 0.14	1.00 ± 0.23	0.91 ± 0.18	0.92 ± 0.21	0.89 ± 0.10
Any adverse neonatal outcome (*n* = 28)	≤ 3	5	8	≤ 3	7	4
Premature delivery (*n* = 19)	≤ 3	≤ 3	4	≤ 3	5	4
Low birth weight (*n* = 19)	≤ 3	4	4	≤ 3	≤ 3	4
SGA (*n* = 9)	≤ 3	≤ 3	**5**	≤ 3	≤ 3	≤ 3

Data presented as absolute numbers and mean ± SD

Gestational age presented as weeks

Small number suppression applied to cells with three or fewer women

*CHD* Congenital heart disease, *CMP* Cardiomyopathy, *VHD* Valvular heart disease, *SD* Standard deviation, *SGA* Small-for-gestational-age

Umbilical artery PI was significantly higher in women with adverse neonatal outcomes than those with healthy neonates (1.05 ± 0.24 PI vs. 0.87 ± 0.13 PI, *p* < 0.0001; Table 1). Umbilical artery PI was significantly higher in women with cardiomyopathy than other heart disease (1.00 ± 0.23 PI vs. 0.88 ± 0.16 PI, *p* = 0.007; Table 3).

Women treated with beta-blockade during pregnancy were significantly more likely to experience adverse neonatal outcomes (64% vs. 40%, *p* = 0.03; Table 2), although this association may not be causal. There was no significant difference between cardiac output in late pregnancy between women who took beta blockers and those who did not (5.61 ± 1.03 L/min vs 5.43 ± 0.99 L/min, *p* = 0.781).

### Echocardiographic assessment of left ventricular systolic function during pregnancy

Pre-pregnancy echocardiograms were available in 34/119 (29%) women. 79/119 (66%) women had an early pregnancy echocardiogram (mean 19 ± 6 weeks, range 2–27 weeks) and 83/119 (70%) women had a late pregnancy echocardiogram (mean 33 ± 3 weeks, range 28–39 weeks).

**Fig.1:**
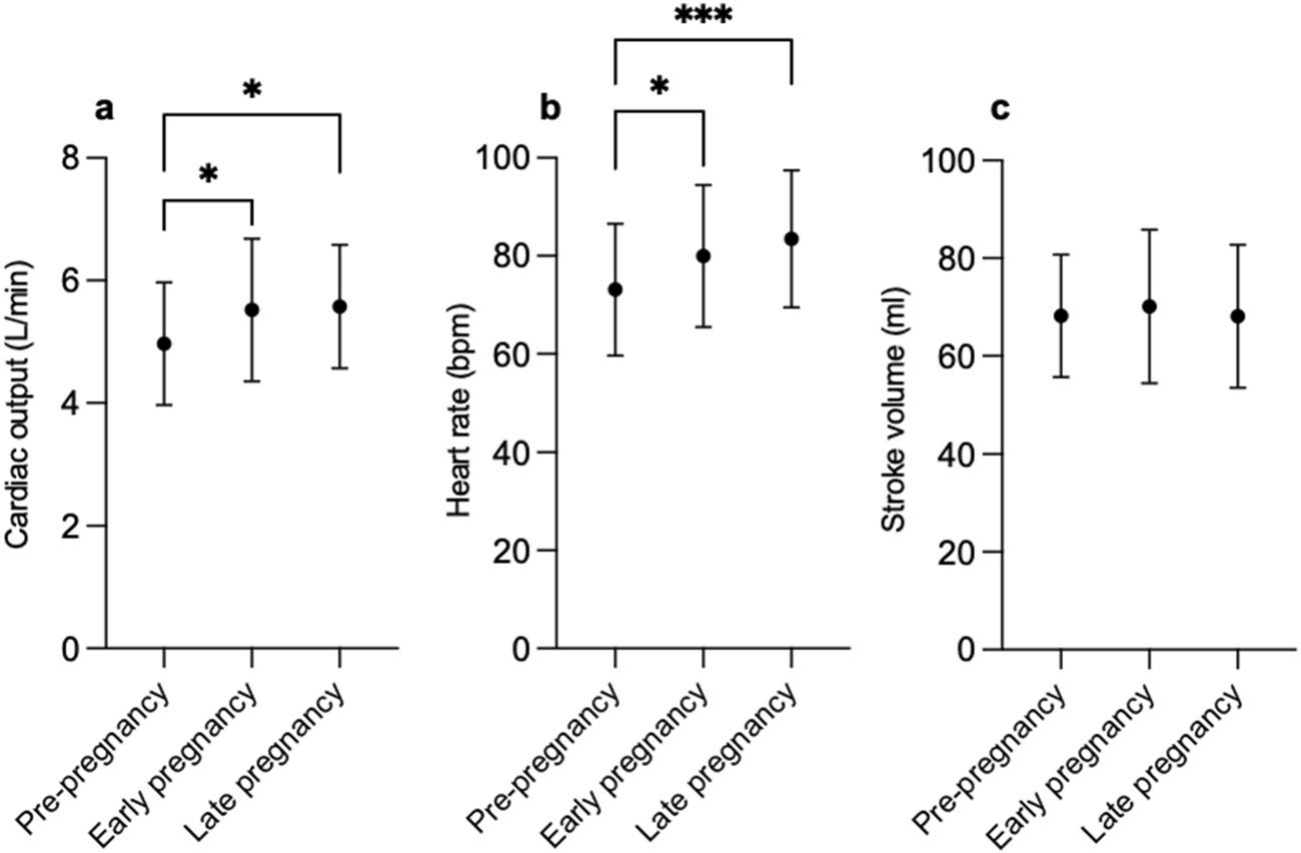
Serial changes in **a** cardiac output, **b** heart rate, and **c** stroke volume during pregnancy. Data presented as mean ± SD. *p < 0.05. ***p < 0.0001

Cardiac output significantly increased during pregnancy. The most significant increase occurred between pre-pregnancy and early pregnancy (4.97 ± 1.00 L/min to 5.52 ± 1.16 L/min, *p* = 0.03), with no significant further increase in late gestation (Fig. 1A). The increase in cardiac output was primarily due to a significant increase in heart rate during pregnancy (73 ± 13 bpm to 83 ± 14 bpm, *p* = 0.0008; Fig. 1B). Overall, there was no significant change in stroke volume across the study time points (*p* = 0.80; Fig. 1C).

**Fig.2:**
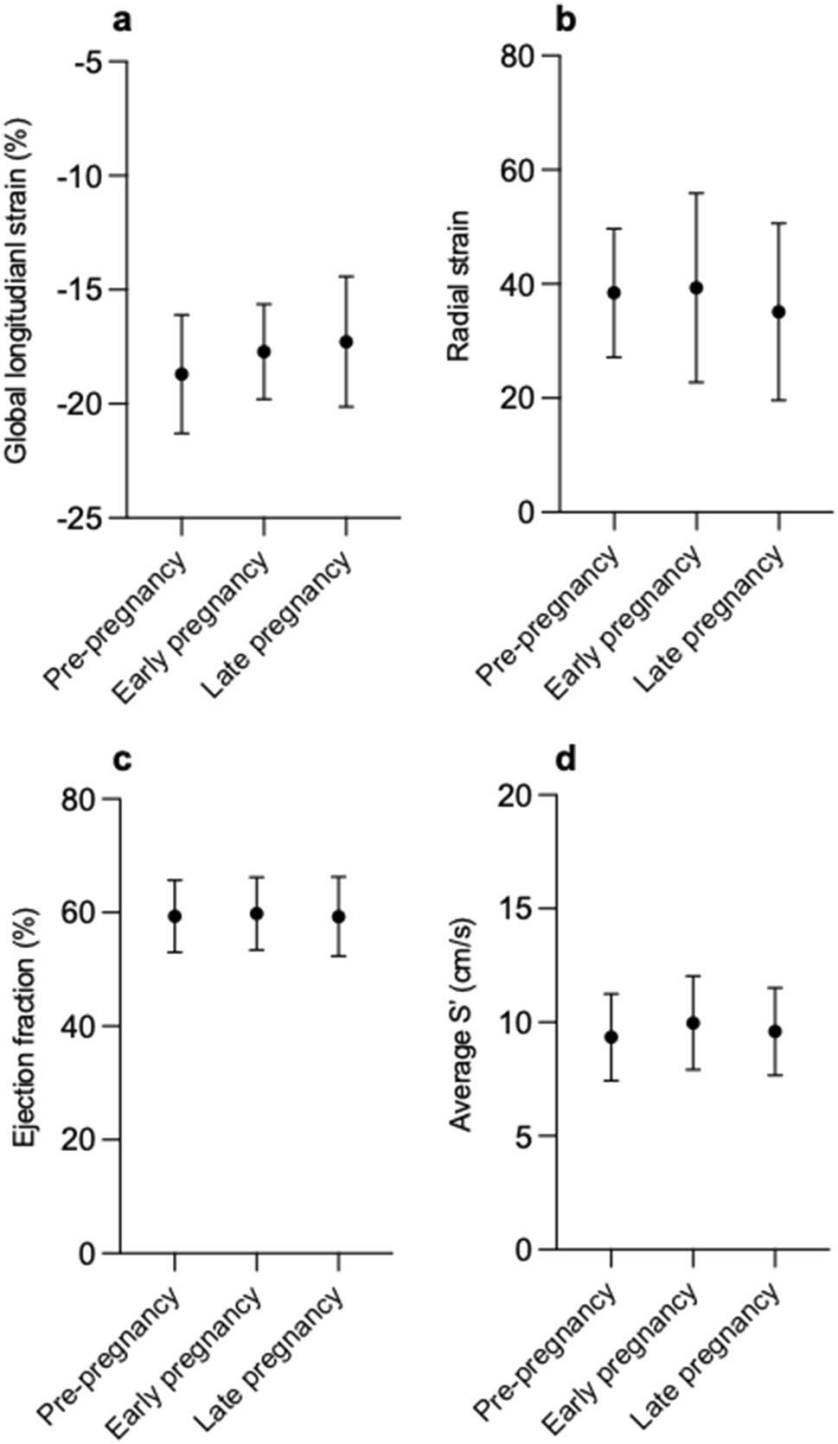
Parameters of left ventricular systolic function pre-pregnancy and during pregnancy in women with cardiac disease. **a** Global longitudinal strain (%). **b** Radial strain at papillary muscle level. **c** Biplane Simpson’s ejection fraction (%). **d** Average S’ (cm/s). Data presented as mean ± SD

During pregnancy GLS decreased from − 18.70% pre-pregnancy to − 17.27% in late pregnancy (Fig. 2A), but this did not meet the threshold for statistical significance (*p* = 0.16). There were no significant changes in RS, EF or Average S’ across pregnancy (Fig. 2B–D).

### Left ventricular systolic function and adverse neonatal outcomes

In early pregnancy, the average S’ was significantly lower in women with adverse neonatal outcomes versus those with healthy neonates (9.07 ± 2.52 cm/s vs. 10.21 ± 1.88 cm/s, *p* = 0.03; Table 4). Other parameters of cardiac function in early pregnancy did not show significant associations with neonatal outcome (Table 4).

**Table 4:** Parameters of cardiac function in early pregnancy in women with cardiac disease with adverse neonatal outcomes and no adverse neonatal outcomes

Parameter	Early pregnancy		*P* value
	Adverse neonatal outcome	No adverse neonatal outcome	
CO (L/min)	5.25 ± 1.16	5.59 ± 1.16	0.32
HR (bpm)	81 ± 18	80 ± 14	0.78
SV (ml)	65.42 ± 13.99	71.32 ± 16.02	0.11
GLS (%)	− 17.66 ± 2.32	− 17.73 ± 2.06	0.94
Radial strain	39.16 ± 18.37	39.40 ± 16.13	0.83
EF biplane (%)	58 ± 8.4	60 ± 5.7	0.79
Average S’ (cm/s)	9.07 ± 2.52	10.21 ± 1.88	**0.03**

Data presented as absolute numbers and mean ± SD

*CO* Cardiac output, *HR* Heart rate, *SV* Stroke volume, *GLS* Global longitudinal strain, *EF* Ejection fraction, *SD* Standard deviation

Average S’ was also significantly reduced in late pregnancy in women with adverse neonatal outcomes (8.67 ± 1.88 cm/s vs. 9.95 ± 1.84 cm/s, *p* = 0.02; Table 5). Additionally, cardiac output was significantly lower in women with adverse neonatal outcomes versus those with healthy neonates (5.11 ± 1.02 L/min vs. 5.77 ± 0.94 L/min, *p* = 0.02; Table 5). Women with adverse neonatal outcomes had a significantly lower stroke volume (61.66 ± 14.56 ml vs. 70.88 ± 13.92, ml *p* = 0.01; Table 5) but no significant difference in heart rate (84 ± 13 bpm vs. 83 ± 15 bpm, *p* = 0.75; Table 5). Other parameters cardiac function in late pregnancy did not show significant associations with neonatal outcome (Table 5).

**Table 5:** Parameters of cardiac function in late pregnancy in women with cardiac disease with adverse neonatal outcomes and no adverse neonatal outcomes

Parameter	Late pregnancy		*P* value
	Adverse neonatal outcome	No adverse neonatal outcome	
CO (L/min)	5.11 ± 1.02	5.77 ± 0.94	**0.01**
HR (bpm)	84 ± 13	83 ± 15	0.75
SV (ml)	61.66 ± 14.56	70.88 ± 13.92	**0.01**
GLS (%)	− 16.87 ± 2.92	− 17.42 ± 2.87	0.61
Radial strain	34.94 ± 15.91	35.16 ± 15.94	0.96
EF biplane (%)	58 ± 6.4	60 ± 7.2	0.41
Average S’ (cm/s)	8.67 ± 1.88	9.95 ± 1.84	**0.02**

Data presented as absolute numbers and mean ± SD

*CO* Cardiac output, *HR* Heart rate, *SV* Stroke volume, *GLS* Global longitudinal strain, *EF* Ejection fraction, *SD* Standard deviation

All significant associations between parameters of left ventricular systolic function and adverse neonatal outcome in early and late pregnancy remained significant following adjustment for beta-blockade in pregnancy.

### Left ventricular systolic function and neonatal birth-weight centile

**Fig.3:**
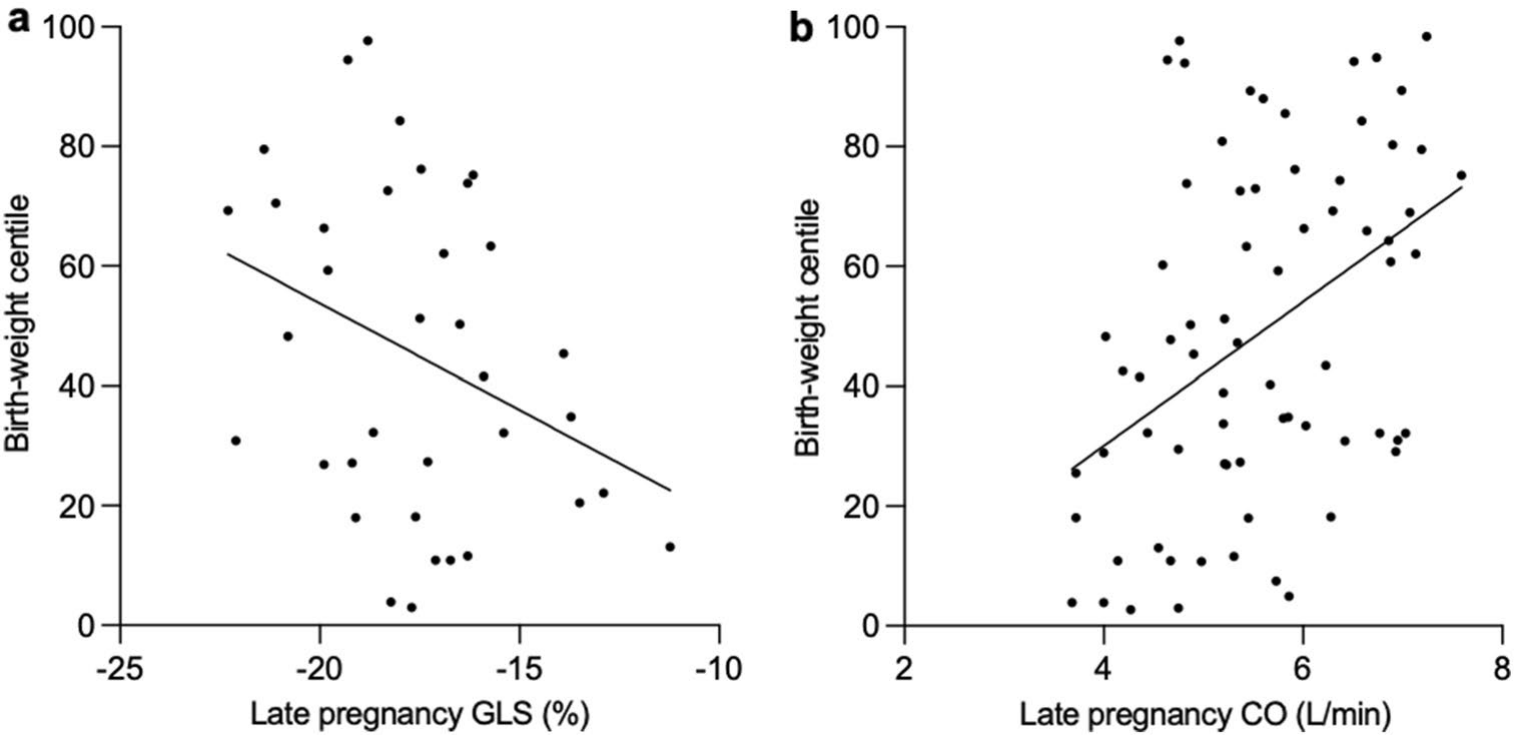
Correlation between birth-weight centile and maternal systolic function. **a** Neonatal birth-weight centile and cardiac output in late pregnancy. **b** Neonatal birth-weight centile and global longitudinal strain in late pregnancy. Abbreviations: GLS: Global longitudinal strain. CO: Cardiac output

In early pregnancy, there were no systolic parameters of left ventricular function that were significantly associated with birth-weight centile. In late pregnancy, there was a significant association between birth-weight centile and both GLS (*R*^2^ = 0.11, *p* = 0.04; Fig. 3A) and cardiac output (*R*^2^ = 0.18, *p* = 0.0002; Fig. 3B). When cardiac output was further investigated, there was a significant positive association between stroke volume and birth-weight centile (*R*^2^ = 0.06, *p* = 0.04), but this did not meet statistical significance for heart rate (*R*^2^ = 0.03, *p* = 0.10). No other systolic parameters of left ventricular function were significantly associated with birth-weight centile in late pregnancy.

### Left ventricular systolic function and utero-placental circulation

**Fig.4:**
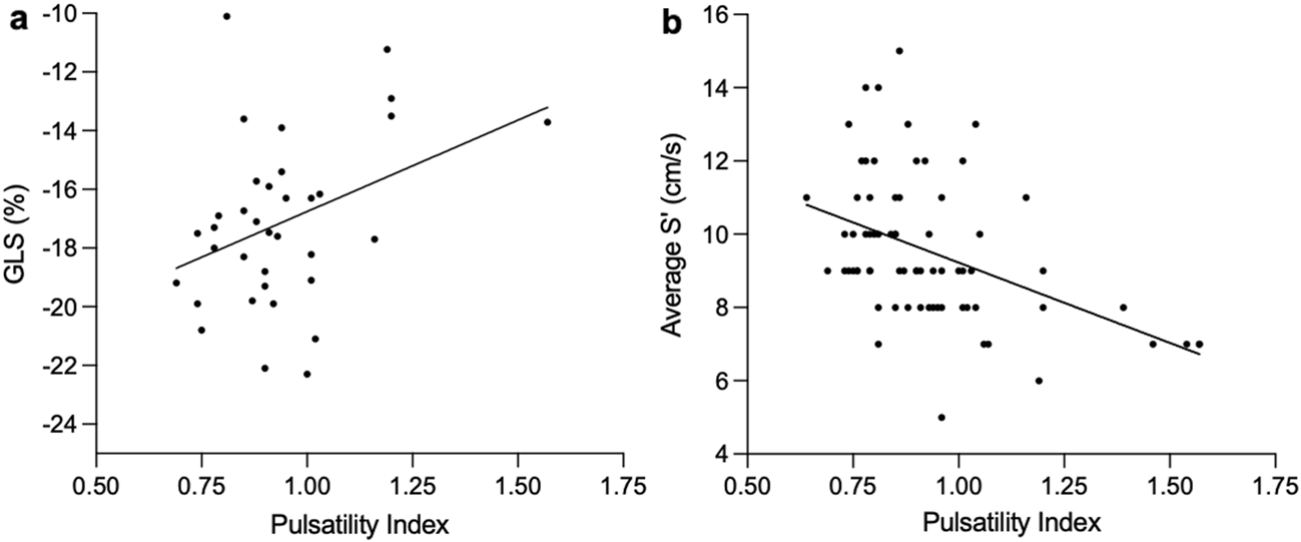
Correlation between pulsatility index and maternal systolic function. **a** Pulsatility index and global longitudinal strain in late pregnancy. **b** Pulsatility index and average S’ in late pregnancy. Abbreviations: GLS: Global longitudinal strain

In early pregnancy, there were no systolic parameters of left ventricular function that were significantly associated with umbilical artery Doppler PI. In late pregnancy, there was a significant association between umbilical artery Doppler PI and both GLS (*R*^2^ = 0.14, *p* = 0.03; Fig. 4A) and average S’ (*R*^2^ = 0.20, *p* < 0.0001; Fig. 4B). Other parameters of left ventricular systolic function in late pregnancy did not show significant associations with umbilical artery Doppler PI.

## Discussion

We show that adverse neonatal outcomes are significantly associated with specific parameters of maternal left ventricular systolic function in both early (average S’) and late (average S’ and cardiac output) pregnancy. Ejection fraction, which is the most commonly assessed measure of left ventricular systolic function [35], was not a significant predictor of adverse neonatal outcome at any stage. Moreover, GLS was also not significantly associated with adverse neonatal outcomes, although lower GLS in late pregnancy was associated with lower birth-weight centile.

Overall, 24% of women in our study experienced adverse neonatal outcomes, which is a similar incidence to other reported cohorts of women with cardiac disease and significantly higher than the expected incidence in a healthy population [20]. In our cohort, 54% of adverse neonatal outcomes occurred in women with valvular heart disease or cardiomyopathy, which is in keeping with the high risks previously reported for these women with these pathologies [14]. Overall, 20% of women with heart disease required hospital admission during pregnancy due to cardiac causes and 66% of these women had an adverse neonatal outcome. These findings highlight the risks associated with pregnancy in women with cardiac disease and the importance of close surveillance by specialist multi-disciplinary teams.

In late pregnancy, we show that cardiac output was significantly lower in women with cardiac disease who went on to have adverse neonatal outcomes than in those with healthy neonates. A similar relationship between cardiac output and neonatal outcome has previously been suggested in women with no known cardiac disease [21]*,* and further evidence suggests that cardiac output may not increase appropriately during pregnancy in women with cardiac disease who have adverse neonatal outcomes [20]. We show significantly reduced stroke volume in late pregnancy in women with adverse neonatal outcomes but no significant difference in heart rate. In cases of adverse outcome, maternal cardiac output may be increasingly limited as pregnancy progresses due to impaired myocardial contractility, suggested by reduced average S' and stroke volume. This is also in keeping with our observation of an excess of adverse outcomes, specifically SGA neonates, in women with cardiomyopathy or valvular lesions compared to other forms of heart disease. The identification of cardiac output that is not appropriately increased by the third trimester should alert the clinician to the increased risk of complications in the neonate, potentially prompting increased surveillance of fetal growth. This finding requires prospective verification in a larger cohort, with appropriate power to determine a threshold for intervention. The association between reduced average S’ and higher umbilical artery Doppler PI in late pregnancy suggests that impaired utero-placental circulation supports the causal link between reduced fetal growth and maternal cardiac disease [17, 36].

We show that by early pregnancy, average S’ was already significantly lower in women who went on to have adverse neonatal outcomes and that this association was maintained into late pregnancy. This suggests decreased longitudinal myocardial velocities in women with heart disease who have adverse neonatal outcomes and supports previous findings that suggest impaired long-axis shortening in women with fetal growth restriction [21]. We investigated the possibility that strain calculation might offer additional clinical benefit in identifying subclinical myocardial dysfunction in women with heart disease and hence increased risk of adverse neonatal outcomes. However, neither GLS nor RS was significantly altered in early or late pregnancy in women with heart disease who had adverse neonatal outcomes compared to those with healthy neonates. Our results suggest that tissue Doppler imaging may be a more appropriate and sensitive parameter to identify myocardial dysfunction to predict adverse outcomes in this cohort of women, and may be a useful parameter to consider in the evaluation of cardiac function in pregnancy. However, there are significant limitations in calculating GLS retrospectively and the possibility that strain could be a useful additional tool to predict outcomes in women with heart disease should not be dismissed without a prospective evaluation.

Our study methodology has significant advantages, including a relatively large [20] and well-phenotyped cohort of women with heart disease, all of whom were managed by a small group of clinicians according to standardised guidelines [10] within a specialised multi-disciplinary service. We also recognise limitations in our study, particularly that women did not have an echocardiogram at all study time points, thus limiting our ability to perform longitudinal assessment of systolic function. Ideally, analysis would have occurred separately in the first and second trimester; however, due to heterogeneity in timing, these were combined as ‘early pregnancy’. Furthermore, a larger sample size would have allowed additional sub-group analyses to be performed, for example more detailed sub-grouping of cardiac pathologies.

## Conclusions

Women with cardiac disease are at increased risk of neonatal complications, in particular fetal growth restriction. Our data suggest a significant association between late pregnancy cardiac output and birth-weight centile. Cardiac output in women with heart disease who have adverse neonatal outcomes is significantly lower in late pregnancy than in those who have healthy neonates, which is likely to impact on the utero-placental circulation. We identify average S’ and cardiac output as parameters of left ventricular systolic function that are significantly associated with risk of adverse neonatal outcomes in women with heart disease. These findings could help refine identification of women whose fetuses are at highest risk of adverse outcome and therefore enable clinicians to target additional fetal surveillance during pregnancy complicated by heart disease.

## Supplementary Information

Below is the link to the electronic supplementary material.

Supplementary file1 (DOCX 14 KB)

Supplementary file2 (DOCX 113 KB)

Supplementary file3 (DOCX 14 KB)

Supplementary file4 (DOCX 14 KB)

Supplementary file5 (DOCX 170 KB)

Supplementary file6 (DOCX 14 KB)

Supplementary file7 (DOCX 16 KB)
