# Supplementary material for: Maternal left ventricular function and adverse neonatal outcomes in women with cardiac disease
Source: Arch Gynecol Obstet. 2022 Jun 3;307(5):1431–9. doi: 10.1007/s00404-022-06635-9 (PMC10110658; doi:10.1007/s00404-022-06635-9)
Supplement: Supplementary file 1 — Supplementary file1 (DOCX 14 KB) [file 404_2022_6635_MOESM1_ESM.docx]

**Online Resource 1** – Table of inclusion criteria

| **Inclusion criteria** |
| --- |
| Pregnant |
| Transthoracic echocardiogram performed at Addenbrooke’s at pre-define timepoints   1. Pre-pregnancy 2. Early pregnancy (0-28 weeks) 3. Late pregnancy (28 weeks-delivery) |
| Delivery at the Rosie Hospital |
| Estimated date of delivery before October 2021 |
