# Supplementary figures and images for: Maternal left ventricular function and adverse neonatal outcomes in women with cardiac disease
Source: Arch Gynecol Obstet. 2022 Jun 3;307(5):1431–9. doi: 10.1007/s00404-022-06635-9 (PMC10110658; doi:10.1007/s00404-022-06635-9)

**Online Resource 2 –** Cohort flow diagram of exclusion


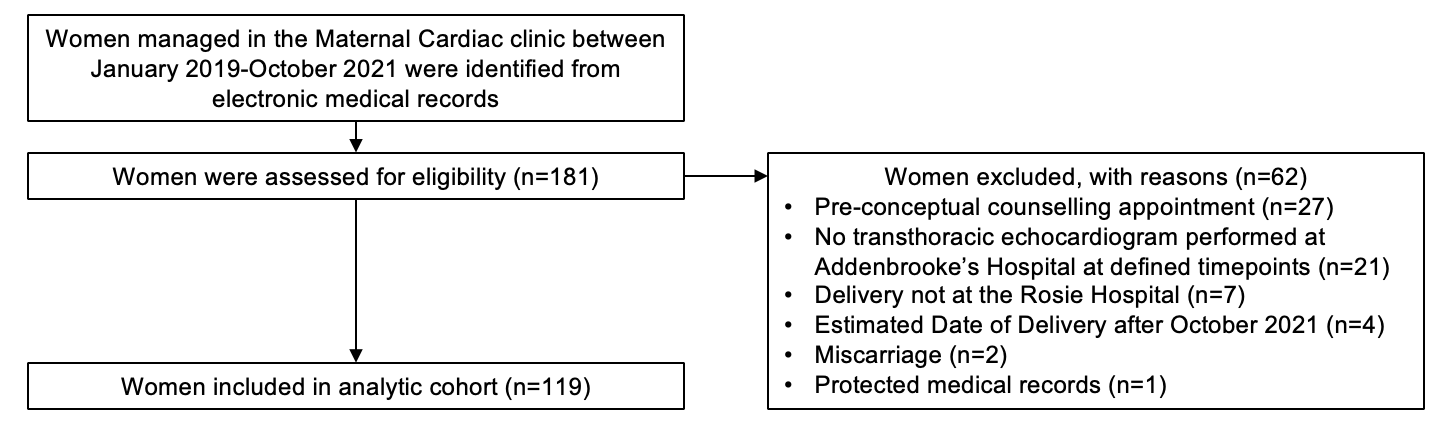

Supplement: Supplementary file 2 — Supplementary file2 (DOCX 113 KB) [file 404_2022_6635_MOESM2_ESM.docx]
