# Supplementary material for: Maternal left ventricular function and adverse neonatal outcomes in women with cardiac disease
Source: Arch Gynecol Obstet. 2022 Jun 3;307(5):1431–9. doi: 10.1007/s00404-022-06635-9 (PMC10110658; doi:10.1007/s00404-022-06635-9)
Supplement: Supplementary file 3 — Supplementary file3 (DOCX 14 KB) [file 404_2022_6635_MOESM3_ESM.docx]

**Online Resource 3 –** Detailed sub-typing of maternal cardiac lesions

| **Cardiac lesion type** | **Cardiac lesion sub-type** |
| --- | --- |
| **Aortopathy**  (n=8) | Aortic root dilatation/coarctation of the aorta (n=8) |
| **Arrhythmia**  (n=24) | SVT/NSVT/pAF (n=16)  Pregnancy-related inappropriate sinus tachycardia (n=8) |
| **Cardiomyopathy**  (n=26) | Peripartum cardiomyopathy (n=5)  Hypertrophic cardiomyopathy/Hypertrophic obstructive cardiomyopathy (n=4)  Dilated cardiomyopathy/chemotherapy-related left ventricular impairment (n=9)  Myocarditis (n=4)  Other (n=4) |
| **Congenital heart disease** (n=28) | Atrial septal defect/Ventricular septal defect (n=12)  Tetralogy of Fallot (n=≤3)  Congenital valve disease (n=7)  Transposition of great arteries (n=3)  Other complex congenital heart disease (n=4) |
| **Valvular heart disease** (n=17) | Mitral valve disease (n=9)  Aortic valve disease (n=5)  Other (n=≤3) |

Table ESM 3: Detailed sub-typing of maternal cardiac lesions. Abbreviations: pAF: Paroxysmal atrial fibrillation, NSVT: Non-sustained ventricular tachycardia, SVT: Supraventricular tachycardia.
