# Supplementary material for: Maternal left ventricular function and adverse neonatal outcomes in women with cardiac disease
Source: Arch Gynecol Obstet. 2022 Jun 3;307(5):1431–9. doi: 10.1007/s00404-022-06635-9 (PMC10110658; doi:10.1007/s00404-022-06635-9)
Supplement: Supplementary file 4 — Supplementary file4 (DOCX 14 KB) [file 404_2022_6635_MOESM4_ESM.docx]

**Online Resource 4** – Ability to calculate Global Longitudinal Strain and Radial Strain

|  | **Total** | **GLS calculated** | **GLS**  **not calculated** | **RS calculated** | **RS**  **not calculated** |
| --- | --- | --- | --- | --- | --- |
| Pre-pregnancy | 34 | 17 (50%) | 17 (50%) | 22 (65%) | 12 (35%) |
| Early pregnancy | 79 | 41 (52%) | 38 (48%) | 63 (80%) | 16 (20%) |
| Late pregnancy | 83 | 38 (46%) | 45 (54%) | 72 (87%) | 11 (13%) |
| Total | 196 | 96 | 100 | 157 | 39 |
