# Supplementary material for: Maternal left ventricular function and adverse neonatal outcomes in women with cardiac disease
Source: Arch Gynecol Obstet. 2022 Jun 3;307(5):1431–9. doi: 10.1007/s00404-022-06635-9 (PMC10110658; doi:10.1007/s00404-022-06635-9)
Supplement: Supplementary file 5 — Supplementary file5 (DOCX 170 KB) [file 404_2022_6635_MOESM5_ESM.docx]

**Online Resource 5** – Calculation of Global Longitudinal Strain and Radial Strain


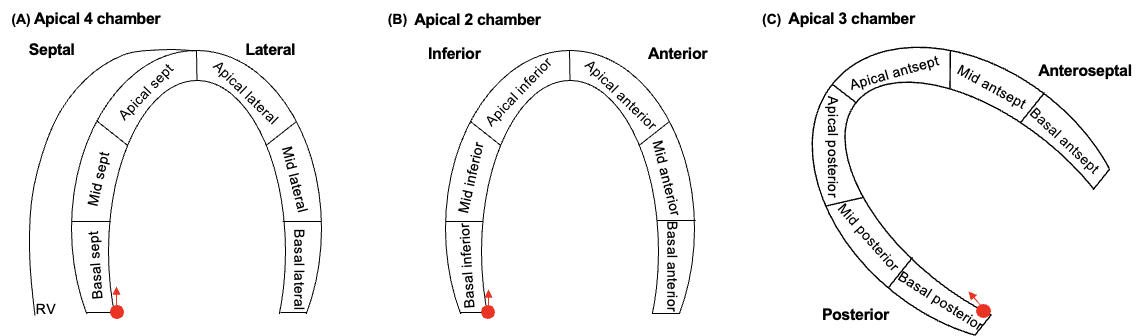


**Figure 1A-C**: Practical demonstration of the method used to calculate global longitudinal strain. Endocardial border tracing should begin in the basal septal, basal inferior and basal posterior points in the apical 4 chamber, apical 2 chamber and apical 3 chamber, respectively. Endocardial border tracing should continue clockwise. Once the endocardial border is traced the region of interest is automatically generated and is then adjusted to ensure that it appropriately fits the endocardial and epicardial border and tracks the myocardium. Abbreviations: Sept: Septal, Antsept: Anteroseptal.


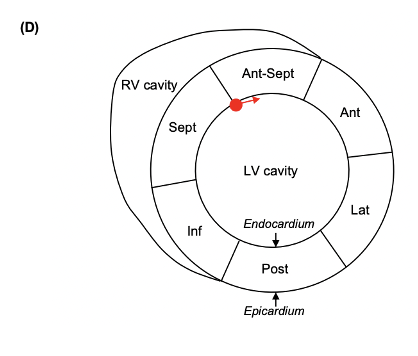


**Figure 1D**: Practical demonstration of the method used to calculate radial strain at the papillary muscle level. Endocardial border tracing should begin in the anteroseptal segment and continue clockwise around to the septal segment. Once the endocardial border is traced the region of interest is automatically generated and is then adjusted to ensure that it appropriately fits the endocardial and epicardial border and tracks the myocardium. Abbreviations: Antsept: Anteroseptal, Ant: Anterior, Lat: Lateral, Post: Posterior, Inf: Inferior, Sept: Septal, LV: Left ventricle, RV: Right ventricle.
