# Supplementary material for: Maternal left ventricular function and adverse neonatal outcomes in women with cardiac disease
Source: Arch Gynecol Obstet. 2022 Jun 3;307(5):1431–9. doi: 10.1007/s00404-022-06635-9 (PMC10110658; doi:10.1007/s00404-022-06635-9)
Supplement: Supplementary file 6 — Supplementary file6 (DOCX 14 KB) [file 404_2022_6635_MOESM6_ESM.docx]

| **Reason GLS not calculated** (n=100) | | **Reason RS not calculated** (n=39) | |
| --- | --- | --- | --- |
| No 2-chamber, 3-chamber or 4-chamber image | 13 (13%) | No parasternal short axis at papillary muscle level | 1 (2.6%) |
| Heart rate too variable | 13 (13%) | Heart rate too high | 2 (5.1%) |
| Frame rate below 40 frames per second | 9 (9%) | Frame rate below 40 frames per second | 3 (7.7%) |
| Poor image quality | 65 (65%) | Poor image quality | 33 (84.6%) |

**Online Resource 6**: Reasons for study exclusion from strain analysis
