# Supplementary material for: Maternal left ventricular function and adverse neonatal outcomes in women with cardiac disease
Source: Arch Gynecol Obstet. 2022 Jun 3;307(5):1431–9. doi: 10.1007/s00404-022-06635-9 (PMC10110658; doi:10.1007/s00404-022-06635-9)
Supplement: Supplementary file 7 — Supplementary file7 (DOCX 16 KB) [file 404_2022_6635_MOESM7_ESM.docx]

| **Maternal characteristic** | | **Aortopathy**  (n=8) | **Arrhythmia**  (n=24) | **CMP**  (n=26) | **CHD**  (n=28) | **VHD**  (n=17) | **Other**  (n=16) |
| --- | --- | --- | --- | --- | --- | --- | --- |
| Ejection fraction  (%) | Pre-pregnancy | 62±6  (n=≤3) | 60±5  (n=5) | 52±9  (n=4) | 61±4  (n=6) | 62±4  (n=5) | 57±11  (n=≤3) |
|  | Early pregnancy | 58±4  (n=5) | 63±3  (n=4) | 52±7  (n=7) | 61±3  (n=13) | 65±5  (n=10) | 62±4  (n=9) |
|  | Late pregnancy | 60±1  (n=≤3) | 63±3  (n=5) | 54±8  (n=18) | 62±7  (n=11) | 61±3  (n=7) | 63±5  (n=9) |
| Heart rate  (bpm) | Pre-pregnancy | 63±11  (n=≤3) | 78±14  (n=7) | 76±17  (n=6) | 75±16  (n=8) | 67±9  (n=6) | 73±8  (n=4) |
|  | Early pregnancy | 78±17  (n=6) | 88±20  (n=10) | 82±13  (n=18) | 74±10  (n=22) | 83±17  (n=11) | 80±13  (n=11) |
|  | Late pregnancy | 82±15  (n=8) | 101±16  (n=10) | 85±12  (n=21) | 78±13  (n=22) | 78±9  (n=11) | 82±9  (n=10) |

**Online Resource 7 –** Charactetrics of maternal cardiac disease

Table ESM 7: Characteristics of maternal cardiac disease. Data presented as mean±SD. Abbreviations: CHD: Congenital heart disease, CMP: Cardiomyopathy, VHD: Valvular heart disease, SD: Standard deviation, SD: Standard deviation.
